# GCMembrane-LLM: An Evidence-Grounded Domain-Specific Large Language Model for Structure–Performance Reasoning in Graphene and Carbon Nanotube Separation Membranes

**DOI:** 10.3390/membranes16060214

**Published:** 2026-06-21

**Authors:** Youyang Liu, Shuhan Liu, Yao He, Ziyi Yan, Yilu Zhao, Xinyu Zhang, Zhen Li, Ning Wei

**Affiliations:** Jiangsu Key Laboratory of Advanced Food Manufacturing Equipment and Technology, Jiangsu Province Engineering Research Center of Micro-Nano Additive and Subtractive Manufacturing, Institute of Advanced Technology, School of Mechanical Engineering, Jiangnan University, Wuxi 214122, China; liuyouyanguu@163.com (Y.L.);

**Keywords:** graphene oxide membranes, carbon nanotube membranes, graphene/CNT hybrid membranes, nanofiltration, desalination, water treatment, membrane fouling, swelling stability, structure–performance relationship, domain-specific large language model

## Abstract

Graphene and carbon nanotube (CNT) membranes are promising for filtration, desalination, and water treatment, yet their performance requires the joint interpretation of their architecture, nanoconfined transport, selectivity, fouling, swelling, defects, stability, and operating conditions. Here, GCMembrane-LLM was developed as an evidence-grounded domain-specific large language model. A curated 582-paper corpus generated 12,208 cleaned membrane-specific question–answer pairs for Low-Rank Adaptation (LoRA)-based supervised fine-tuning of Llama-3.1-8B-Instruct, and retrieval-augmented generation provided article-title and page-level traceability. GCMembraneBench included 100 application-oriented questions on graphene oxide (GO) membranes, CNT membranes, GO/CNT hybrids, and cross-material reasoning. Under direct answering without retrieval context, the anonymized and shuffled automatic evaluation showed that GCMembrane-LLM achieved a mean weighted score of 4.237/5.0, exceeding Llama-3.1-8B-Instruct and Doubao-1.5-lite. A stratified 30-question blinded manual assessment showed the same ranking. The application cases further yielded membrane science conclusions: CNT-assisted GO/CNT transport should be evaluated with dispersion, interfacial compatibility, defects, and stability; GO desalination depends on swelling control, interlayer spacing, and defect suppression; and CNT high flux requires joint examination of pore diameter, entrance chemistry, hydration barriers, ion rejection, and operating conditions. GCMembrane-LLM supports source-traceable evidence organization and preliminary hypothesis formulation before experimental validation.

## 1. Introduction

Membrane-based separation has become an important platform for desalination, water purification, wastewater treatment, resource recovery, and selective ion or molecular separation [1,2,3,4]. For practical applications, membrane performance cannot be evaluated solely by water flux. Effective membranes must also maintain selectivity or rejection, suppress fouling and swelling, tolerate realistic feed compositions and operating pressures, and preserve stable performance during long-term operation [3,4,5,6,7]. Accordingly, membrane development requires the integrated interpretation of how membrane structure, transport behavior, testing conditions, and application constraints jointly determine separation outcomes. For membrane researchers, a reported performance enhancement is meaningful only when it is supported by evidence linking material architecture, nanochannel transport, selectivity, antifouling behavior, swelling stability, operating conditions, and long-term application limitations.

Among emerging membrane materials, graphene- and carbon nanotube (CNT)-based membranes have attracted substantial attention because of their distinctive nanoconfined transport characteristics. Graphene-derived membranes, particularly graphene oxide (GO) laminates, contain two-dimensional nanochannels with tunable interlayer spacing and abundant oxygen-containing functional groups [8,9,10,11,12,13,14,15,16], allowing the regulation of water transport, molecular sieving, and ion selectivity [17,18,19,20,21,22,23,24]. CNT-based membranes provide atomically smooth and relatively hydrophobic inner walls [25,26,27,28,29], which can promote rapid, low-friction water transport [30,31,32,33]. Hybrid graphene/CNT architectures further combine the laminar selectivity of graphene-derived frameworks with CNT-enabled spacing regulation and transport acceleration [34,35,36,37,38]. Accordingly, graphene/CNT membranes have shown considerable potential in relation to desalination, nanofiltration, ultrafiltration, dye removal, heavy metal removal, wastewater treatment, and antifouling membrane design [13,16,32,33,36,37,38]. In this work, graphene/CNT membranes are discussed across pressure-driven membrane processes, including nanofiltration, ultrafiltration, reverse osmosis-related desalination, and broader water treatment applications, with distinctions retained among GO laminates, CNT membranes, and graphene/CNT hybrid membranes. Across this literature, however, performance claims are often difficult to compare directly because flux, permeance, rejection, fouling resistance, swelling behavior, and stability are reported under different membrane architectures, thicknesses, feed compositions, operating pressures, testing durations, and reporting formats. This evidence fragmentation creates a central challenge for graphene/CNT membrane research, where reported performance must be interpreted through coupled structure–transport–condition relationships rather than isolated performance metrics.

Despite these advantages, the practical design of graphene/CNT membranes remains a complex multi-objective challenge [39]. In GO laminates, increased interlayer spacing may enhance water permeance, but salt rejection can be weakened when swelling, structural defects, or nonselective leakage pathways are introduced [17,18,19,20,22,39]. CNT incorporation may reduce transport resistance; however, excessive loading, poor dispersion, or interfacial void formation can compromise selectivity and structural integrity [32,33,34,35,36]. Surface chemical regulation may improve ion discrimination under specific solution conditions, while fouling resistance, mechanical robustness, and long-term chemical stability may also be affected [14,15,16,18,19,20,37,38]. Moreover, the mechanisms underlying high separation performance remain under active discussion, including slip-flow-dominated transport in CNT nanochannels, capillary-driven or interfacial-friction-regulated transport in GO galleries, and the roles of defects, hydration effects, and functional groups in governing permselectivity [22,25,26,27,28,29,30]. Evidence-grounded reasoning is therefore essential, because a reported high flux may arise from genuine low-resistance nanoconfined transport, enlarged nonselective defects, swelling-induced leakage, reduced membrane thickness, or differences in operating pressure and feed conditions. Therefore, the central problem addressed in this study is how to interpret reported graphene/CNT membrane performance under coupled structure–transport–condition constraints, where flux or permeance, selectivity or rejection, fouling resistance, swelling stability, defect control, operating conditions, and long-term durability must be evaluated together rather than as isolated performance indicators.

Another major challenge is that application-relevant knowledge remains dispersed across a rapidly expanding and heterogeneous literature landscape. With the increasing publication volume in graphene/CNT membrane research, the manual synthesis of structure–performance evidence has become increasingly difficult. Relevant information is frequently distributed across abstracts, experimental sections, tables, figure captions, Supplementary Materials, and discussion paragraphs. Reported membrane performances are also obtained under different operating pressures, feed concentrations, membrane thicknesses, testing areas, and operation durations, which complicates direct comparison across studies [2,3,4,5,6,7,39]. Conventional review articles provide valuable summaries, but they are static in format and cannot respond interactively to design-oriented questions. Structured materials informatics and machine learning approaches can support performance prediction when standardized numerical datasets are available [40,41,42,43,44]; however, many insights related to membrane structure, transport behavior, fouling resistance, swelling stability, defect formation, and application conditions remain embedded in the unstructured full-text literature. General-purpose large language models (LLMs) offer a potential route for organizing such unstructured scientific knowledge, although they are not specifically optimized for the terminology, material distinctions, and coupled design logic of graphene/CNT membrane separations. Recent domain-specific scientific language models have shown that curated domain corpora can improve specialized information extraction and reasoning in materials research [45,46], with GrapheneChat providing a relevant example in graphene research [46]. Nevertheless, graphene/CNT separation membranes require application-oriented reasoning that jointly considers membrane architecture, transport pathways, selectivity, fouling, swelling, operating conditions, and stability constraints.

**Figure 1:**
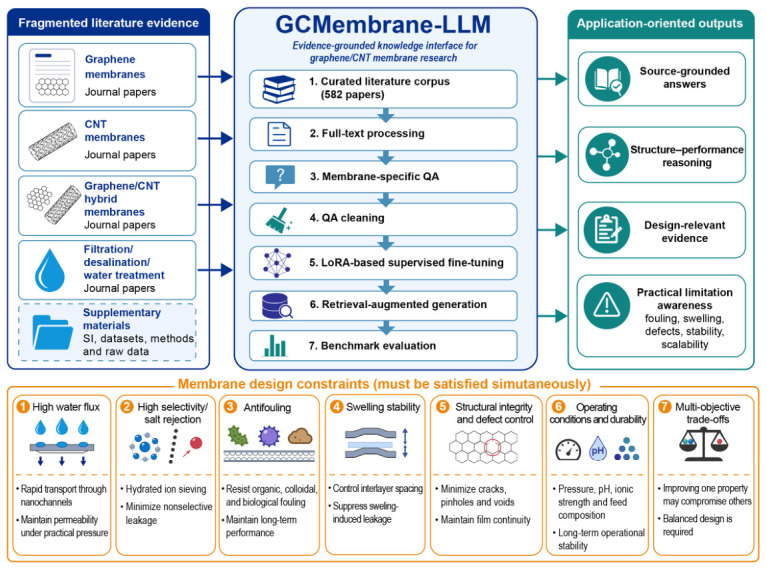
Conceptual framework of GCMembrane-LLM for application-oriented graphene/CNT membrane research. The framework links the fragmented graphene/CNT membrane literature with the main model development workflow, including corpus construction, full-text processing, membrane-specific QA generation, data cleaning, Low-Rank Adaptation (LoRA)-based supervised fine-tuning, retrieval-augmented generation (RAG), and benchmark evaluation. The outputs include source-grounded answers, structure–performance reasoning, design-relevant evidence organization, and practical limitation awareness under coupled membrane-design constraints such as flux, selectivity, fouling, swelling, defect control, operating conditions, and durability.

To address these challenges, GCMembrane-LLM was developed as an evidence-grounded, domain-specific LLM for application-oriented graphene/CNT membrane research. The overall conceptual framework is illustrated in Figure 1. The model was developed from Llama-3.1-8B-Instruct and adapted through Low-Rank Adaptation (LoRA)-based supervised fine-tuning (SFT) using a curated 582-paper corpus and membrane-specific question–answer (QA) data [47,48,49]. Retrieval-augmented generation (RAG) was further integrated to provide article-title and page-level source traceability and to constrain answer generation with the retrieved literature evidence [50], while ChromaDB v1.5.8 was used as the persistent vector database for embedding storage and similarity-based retrieval [51]. GCMembrane-LLM is positioned as a source-grounded reasoning interface for evidence-grounded interpretation, structure–mechanism–condition reasoning, and pre-experimental hypothesis formulation in graphene/CNT membrane research. Its intended role is to organize fragmented literature evidence and examine whether reported structure–performance claims are consistent with membrane architecture, transport mechanism, testing conditions, and application limitations. Autonomous membrane design and the replacement of transport experiments are beyond the scope of this framework. The direct-answer capability of GCMembrane-LLM was evaluated using GCMembraneBench, while source-grounded traceability and qualitative membrane science reasoning were examined through representative RAG-based cases across GO/CNT composite membranes, GO laminates, and CNT membranes.

## 2. Materials and Methods

### 2.1. Literature Corpus Construction

A domain-specific literature corpus was constructed to support the development of GCMembrane-LLM. The target scope covered graphene/CNT membranes for filtration, desalination, water purification, ion separation, nanofiltration, ultrafiltration, wastewater treatment, and related aqueous separation applications [13,21,32,33,37,38,39]. The initial retrieval was performed through the OpenAlex application programming interface (API; accessed on 18 June 2026) using a predefined keyword strategy that covered graphene–CNT composite membranes, graphene/CNT composite membranes, GO–CNT membranes, GO–CNT hybrid membranes, graphene membranes, CNT membranes, GO membranes, and membrane-separation terms, including desalination, water treatment, reverse osmosis, forward osmosis, salt rejection, molecular sieving, ion separation, and water permeation. The search was conducted on 3 April 2026. To improve reproducibility and define a clear retrieval boundary, only Digital Object Identifier (DOI)-bearing journal articles were included. The final corpus contained papers published between 2014 and 2026.

The retrieval workflow exported article titles, DOIs, publication years, citation counts, open-access status, journal names, and reconstructed abstracts. During the initial retrieval stage, non-target research fields were excluded, including photocatalysis, batteries, supercapacitors, sensors, electrodes, biomedical imaging or therapy, solar cells, actuators, ceramics, foams, triboelectric devices, piezoresistive devices, MXene membranes, and MoS_2_-related systems. The initial search was deliberately broad to reduce the risk of excluding potentially relevant graphene/CNT membrane studies before secondary screening.

**Figure 2:**
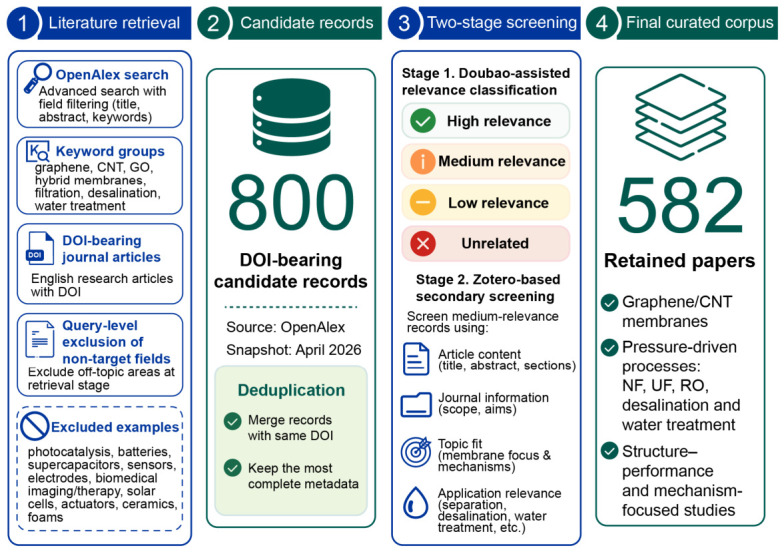
Literature retrieval, screening, and corpus construction workflow for GCMembrane-LLM. Candidate records were retrieved from OpenAlex using a predefined graphene/CNT membrane keyword strategy and restricted to Digital Object Identifier (DOI)-bearing journal articles. After the exclusion of non-target research fields, Doubao-assisted relevance classification through the Volcengine application programming interface (API), and Zotero-based secondary screening, 582 papers were retained from 800 candidate records as the final curated corpus for full-text extraction, question–answer generation, supervised fine-tuning, retrieval-augmented generation, and model evaluation.

After retrieval, a two-stage screening procedure was performed. First, a Doubao-powered screening agent, accessed through the Volcengine API (accessed on 18 June 2026), was used to classify the collected records into high, medium, low, and unrelated relevance categories according to their fit with graphene/CNT membrane separations. Second, DOI-based records were imported into Zotero 7.0.32 for manual secondary screening, during which article content, journal information, topic relevance, and consistency with the target membrane application scope were further examined. After this two-stage screening process, 582 papers were retained as the final corpus. The retained corpus was used for subsequent full-text extraction, QA generation, SFT, RAG, and model evaluation. The overall literature retrieval, screening, and corpus construction workflow is summarized in Figure 2.

### 2.2. Full-Text Processing and Metadata Traceability

The selected literature files were uploaded to Alibaba Cloud Object Storage Service (OSS; accessed on 18 June 2026) and mounted in an Alibaba Cloud Data Science Workshop (DSW; accessed on 18 June 2026) environment for stable access and batch processing. For QA data construction, full-text content was extracted from the Portable Document Format (PDF) files using custom Python scripts based on PyMuPDF 1.27.2.2 in a Python 3.11.14 environment. The extracted text was cleaned by removing identifiable reference list sections, repairing line-break hyphenation, reducing excessive whitespace, and truncating overly long fragments to control the input length for batch inference. This preprocessing reduced parsing noise and generated standardized paper-level text inputs for membrane-specific QA generation.

For RAG, page-level traceability was maintained through a separate chunk-construction workflow. In this workflow, each PDF page was processed together with its metadata, including the article title, PDF filename, page number, OSS storage path, and document identifier. These metadata fields were assigned to the corresponding text chunks and retained during vector indexing, retrieval, answer generation, and final source display. This design enabled GCMembrane-LLM to return generated answers with article-title and page-level source information, thereby improving the transparency and verifiability of evidence-grounded outputs.

Because the present workflow relied mainly on extracted page-level text, the corpus representation was designed primarily for traceable qualitative evidence organization. Article-title and page-level metadata were therefore retained for each retrieved chunk, allowing the original literature page to be inspected when numerical performance values, operating conditions, table-based data, or figure-based evidence required verification. Numerical information presented in tables, plotted curves, figure panels, and supplementary datasets was therefore handled through page-level traceability rather than structured numerical extraction.

### 2.3. Membrane-Specific Question–Answer Generation and Data Cleaning

The cleaned full-text corpus was converted into JavaScript Object Notation Lines (JSONL) batch-task files according to the input format required by Alibaba Cloud Model Studio (accessed on 18 June 2026). A domain-specific prompt was designed to guide professional QA generation from each paper. The prompt focused on graphene membranes, CNT membranes, graphene/CNT hybrid membranes, nanochannel structures, water transport, salt rejection, ion selectivity, antifouling behavior, membrane stability, scalable preparation, specific energy consumption, and application-oriented membrane design.

The generated JSONL batch-task files were uploaded to the Alibaba Cloud Model Studio console and submitted as batch inference tasks through the Qwen Batch application programming interface (API; accessed on 18 June 2026) using Qwen-Max (accessed on 18 June 2026). For each processed paper, Qwen-Max generated membrane-specific QA pairs covering membrane mechanisms, structure–performance relationships, performance metrics, application scenarios, and practical limitations. The raw batch outputs were stored as nested JSONL response files, and the generated QA pairs were extracted from the model-response field and converted into the supervised fine-tuning format required by LLaMA-Factory v0.9.5.dev0 (commit fa09c01), which consisted of instruction, input, and output fields. During format conversion and preliminary cleaning, empty records, malformed responses, duplicated questions, and overly short answers were removed.

After the preliminary cleaning, 28,563 membrane-specific QA pairs were retained as the candidate dataset. A subsequent model-assisted quality-control step was conducted using Qwen-3.5-plus (accessed on 18 June 2026) as an automatic domain-oriented judge. The judge prompt specified a strict specialist role in graphene/CNT membranes and membrane separations. Each QA pair was assessed for relevance, information density, scientific plausibility, and logical consistency between the question and the answer. To improve judgment stability, the temperature was set to 0.1, and the model output was restricted to either “KEEP” or “DISCARD”.

Only QA pairs labeled “KEEP” were retained for SFT. After the complete cleaning and quality-control process, 12,208 QA pairs remained in the final membrane-specific SFT dataset, corresponding to a retention rate of 42.74%. Rule-based multi-label topic analysis was then performed to inspect the thematic coverage of the cleaned dataset, including membrane structure, transport mechanism, separation performance, fouling/antifouling, swelling/stability, operating conditions, and scale-up or practical limitations.

**Figure 3:**
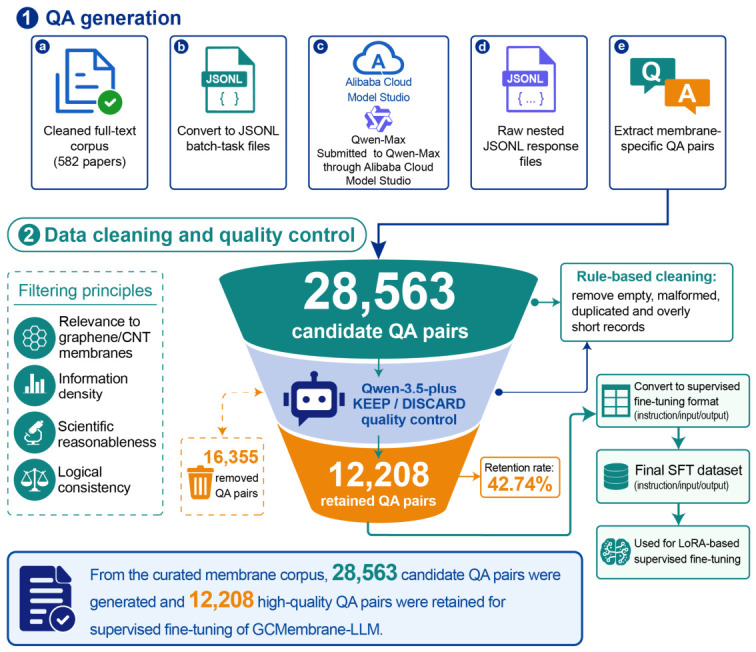
Workflow of membrane-specific question–answer (QA) generation, data cleaning, and quality control for GCMembrane-LLM. The QA-generation stage comprises (a) preparation of the cleaned full-text corpus, (b) conversion into JSONL batch-task files, (c) Qwen-Max batch inference through Alibaba Cloud Model Studio, (d) storage of raw nested JSONL response files, and (e) extraction of membrane-specific QA pairs. A total of 28,563 candidate QA pairs were subjected to rule-based cleaning and Qwen-3.5-plus KEEP/DISCARD quality control; 12,208 retained pairs were then converted into supervised fine-tuning format and used for LoRA-based supervised fine-tuning.

Training data quality was further examined through a manual spot-check of 100 randomly sampled QA pairs from the final cleaned SFT dataset. The sampled records were evaluated using four criteria: topic relevance to graphene/CNT membrane separations, scientific plausibility, QA consistency, and correct distinction among GO laminates, CNT membranes, and GO/CNT hybrid membranes. Records were labeled KEEP when all four criteria were satisfied and FLAG when weak relevance, insufficient graphene/CNT membrane specificity, incomplete membrane science context, or a material–system mismatch was identified. The workflow of membrane-specific QA generation and data cleaning is summarized in Figure 3.

### 2.4. Supervised Fine-Tuning of GCMembrane-LLM

GCMembrane-LLM was developed through SFT of Llama-3.1-8B-Instruct [47]. The cleaned membrane-specific QA dataset was reformatted according to the LLaMA-Factory SFT format, which includes the “instruction”, “input”, and “output” fields [49]. After format validation, 12,208 valid records were retained in the final SFT dataset. Before training, the JavaScript Object Notation Lines (JSONL) file was checked for structural consistency and registered in the LLaMA-Factory dataset configuration file.

The SFT was designed to adapt the general instruction-following capability of the base model to membrane-specific terminology, mechanistic interpretation, structure–performance relationships, and application-oriented reasoning in graphene/CNT membrane research. The training data covered graphene membranes, CNT membranes, graphene/CNT hybrid membranes, nanoconfined water transport, salt rejection, ion selectivity, membrane fouling, swelling control, structural stability, scalable preparation, and filtration and desalination applications. This domain-adaptation strategy is consistent with recent progress in specialized scientific LLMs for graphene research, in which curated domain data have been shown to improve expert-level knowledge retrieval and reasoning [46].

Fine-tuning was conducted with LoRA in LLaMA-Factory during the supervised fine-tuning stage [48,49]. The LoRA configuration used a rank of r = 8, a scaling factor of α = 16, and a dropout rate of 0.0. All LoRA-supported trainable projection modules were targeted, including q_proj, k_proj, v_proj, o_proj, gate_proj, up_proj, and down_proj. The maximum sequence length was set to 2048 tokens. Training was performed for three epochs with a per-device training batch size of two and gradient accumulation over four steps, corresponding to an effective batch size of eight. The learning rate was set to 5 × 10^−5^, and a cosine learning-rate scheduler with a warm-up ratio of 0.1 was applied. Additionally, 16-bit floating-point (FP16) precision, the paged AdamW 8-bit optimizer, automatic flash attention configuration, and gradient checkpointing were used to improve memory efficiency. After training, the LoRA checkpoint was saved in the LLaMA-Factory output directory and used as the domain-adapted GCMembrane-LLM for subsequent inference and evaluation. The core supervised fine-tuning configuration is summarized in Table 1.

### 2.5. Retrieval-Augmented Generation

**Figure 4:**
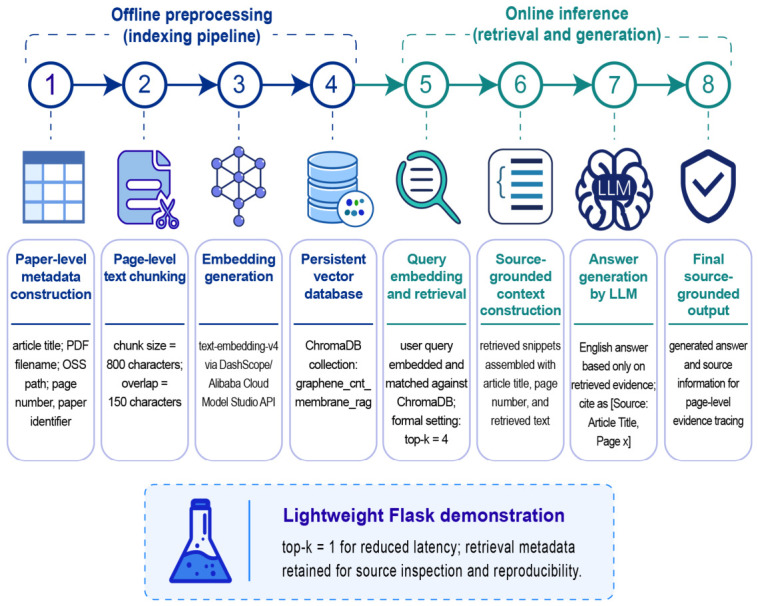
Retrieval-augmented generation (RAG) workflow for source-grounded inference in GCMembrane-LLM. Numbers 1–8 denote the sequential stages from offline corpus indexing to online retrieval, answer generation, and final source-grounded output. Page-level chunks with preserved metadata were embedded using text-embedding-v4 and indexed in ChromaDB; during inference, top-k = 4 retrieved snippets were assembled as evidence for answer generation, and the final output included the generated answer with article-title and page-level source information. For the lightweight demonstration implemented with Flask v3.1.3, top-k was reduced to 1 to decrease response latency.

A RAG module was implemented to provide source traceability and to constrain answer generation with the retrieved literature evidence [50]. As illustrated in Figure 4, the RAG workflow for source-grounded inference consisted of two stages: offline corpus indexing and online retrieval-based answer generation. During offline indexing, a paper-level metadata table was constructed from the curated literature corpus, and each paper was assigned a unique identifier. The article title, PDF filename, and OSS path were retained as paper-level metadata, whereas page number was recorded during page-level PDF text extraction and preserved throughout chunking, vector indexing, retrieval, and final source display.

During PDF processing, page-level text was segmented into chunks with a chunk size of 800 characters and an overlap of 150 characters. Each chunk retained the corresponding paper identifier, article title, PDF filename, OSS path, and page number. The processed chunks were exported in JSONL format and embedded using text-embedding-v4 (accessed on 18 June 2026) through the DashScope API of Alibaba Cloud Model Studio. The resulting embeddings and metadata were stored in a persistent ChromaDB vector database, and retrieval was performed by nearest-neighbor vector similarity search following general vector database principles [51].

During formal RAG-based inference, the user query was embedded using the same embedding model and searched against the ChromaDB vector database with top-k = 4. The retrieved snippets were assembled into a source-grounded context containing the article title, page number, and retrieved text, and this context was inserted into the prompt for the supervised fine-tuned Llama-3.1-8B-Instruct model [47]. The prompt required the model to answer in English, use only the retrieved evidence, avoid unsupported claims, and cite sources in the format “[Source: Article Title, Page X]”. The final output contained the generated answer together with the corresponding article-title and page-level source information.

Each predefined membrane science case used several semantically related retrieval queries to obtain candidate records and improve evidence coverage. These records were screened using predefined keyword-relevance rules, retained metadata, and title-level exclusion criteria for clearly mismatched results. For each case, up to four selected retrieval records were used to construct the source-grounded prompt. The generated answer was organized into three sections, with at least one article-title and page-level citation required in each section.

To further quantify the retrieval behavior of the RAG module, a supplementary small-scale retrieval relevance evaluation was conducted using 30 membrane science queries across six categories, including GO swelling and salt rejection, CNT transport and ion exclusion, GO/CNT hybrid transport, fouling and antifouling, operating conditions, and practical limitations. For each query, the top four retrieved chunks were exported with article title, page number, retrieval rank, vector distance, and retrieved text. Each retrieved chunk was manually labeled as relevant or irrelevant according to whether it directly supported the query. Hit@1, Hit@4, Precision@4, and mean reciprocal rank (MRR) were then calculated. This evaluation quantified retrieval relevance at the retrieved chunk level, while sentence-level citation faithfulness, citation accuracy, and unsupported claim rate were treated as remaining evaluation boundaries.

### 2.6. Benchmark Design, Model Comparison, and Statistical Analysis

Comparative model performance was evaluated using GCMembraneBench, an internal application-oriented benchmark comprising 100 English questions. The benchmark was designed to assess direct-answer capability for graphene/CNT membrane separation tasks, including domain relevance, structure–performance reasoning, practical usefulness, technical accuracy, and practical limitation awareness. The questions were organized into six categories: GO membrane applications and mechanisms, CNT membrane transport and separation mechanisms, GO/CNT hybrid membrane design, cross-material structure–performance evaluation, application condition and performance evaluation, and application-oriented design and troubleshooting. These categories contained 20, 20, 20, 15, 15, and 10 questions, respectively.

Question-level textual isolation was examined before model evaluation by screening all 100 benchmark questions against the complete set of 12,208 retained SFT QA records. Normalized exact matching, character n-gram term frequency–inverse document frequency (TF-IDF) cosine similarity, SequenceMatcher similarity, and keyword Jaccard overlap were calculated. No exact duplicates or very high textual overlap cases were identified, whereas two questions showed partial textual overlap and 98 questions showed no direct textual duplication. Because the benchmark records did not contain source paper identifiers or DOI fields, this analysis assessed direct textual duplication and high wording overlap with the retained SFT QA records. The benchmark questions were designed as application-oriented prompts for direct-answer evaluation rather than source-specific retrieval questions derived from individual papers, and no retrieval context was provided during benchmark answer generation. Therefore, paper-level source isolation and semantic leakage were outside the scope of this check.

Three models were evaluated using the same 100 benchmark questions: GCMembrane-LLM, the original Llama-3.1-8B-Instruct baseline, and Doubao-1.5-lite accessed through an online inference endpoint. GCMembrane-LLM and the original Llama-3.1-8B-Instruct baseline were evaluated on the same development instance. For GCMembrane-LLM, the Llama-3.1-8B-Instruct base checkpoint was loaded together with the trained LoRA adapter, whereas the baseline model was loaded without the LoRA adapter. Doubao-1.5-lite was evaluated through a Volcengine Ark inference endpoint (accessed on 18 June 2026). Each model generated answers to the same benchmark questions, and all outputs were saved using a unified schema including question identifier, category, subtopic, difficulty label, model name, generated answer, run status, and response time. The present comparison was limited to the original base model and one general online model endpoint because no directly comparable graphene/CNT membrane-specific generative LLM baseline was available in the present study, and additional scientific, chemistry, materials-domain, or membrane-engineering LLMs could not be consistently deployed under the same evaluation environment. Therefore, the evaluation was designed to examine whether graphene/CNT membrane-specific SFT improved performance relative to the selected baselines, rather than to claim comprehensive superiority over all scientific or materials-domain language models.

Benchmark answer generation was performed without retrieval context so that GCMembraneBench assessed the direct-answer capability associated with domain-specific SFT, while retrieval-augmented source grounding was examined separately through the RAG source-tracing example and representative membrane science cases. Qwen-3.5-plus was used as the automatic judge model for comparative evaluation, following the general use of LLM-as-a-judge protocols while recognizing their potential evaluation biases [52]. To reduce identity and position bias, the complete 100-question automatic evaluation was rerun using anonymized answer labels and shuffled answer order. For each benchmark question, the three model responses were presented to the judge only as Answer A, Answer B, and Answer C in randomized order, and the private answer-label-to-model mapping was used only after scoring to aggregate model-level results. The judge temperature was set to zero, and the maximum output length was set to 1600 tokens.

Each answer was scored on a 1-to-5 scale across six dimensions: domain relevance, practical usefulness, structure–performance reasoning, technical accuracy, practical limitation awareness, and clarity/conciseness. The first five dimensions were used to calculate the final weighted overall score because they directly reflected the intended role of GCMembrane-LLM as an application-oriented membrane research assistant. Clarity/conciseness was retained as an auxiliary diagnostic indicator and was excluded from the final weighted score calculation.

The final weighted overall score was calculated as follows:

Overall Score = 0.278D + 0.222A + 0.222S + 0.167T + 0.111L

where D, A, S, T, and L denote domain relevance, practical usefulness, structure–performance reasoning, technical accuracy, and practical limitation awareness, respectively. The weights were obtained by normalizing the five domain-relevant dimensions after excluding clarity/conciseness. Higher weights were assigned to domain relevance, practical usefulness, and structure–performance reasoning because these dimensions were most closely aligned with the application-oriented purpose of GCMembrane-LLM.

Model-level mean scores, dimension-level mean scores, and question-level paired differences were calculated from the automatic judge outputs. Bootstrap resampling with 10,000 iterations was used to estimate 95% confidence intervals for model-level weighted scores and paired score differences. Paired differences were calculated at the question level by subtracting the score of each baseline model from the GCMembrane-LLM score for the same benchmark question. The robustness of the model ranking to the weighting scheme was further assessed by recalculating the overall scores under equal weights and alternative dimension-emphasis schemes that prioritized technical accuracy, practical usefulness, or structure–performance reasoning.

The evaluation protocol defined GCMembraneBench as an internal controlled benchmark within the graphene/CNT membrane domain. Model-level mean scores, dimension-level mean scores, question-level paired differences, bootstrap confidence intervals, and fractional win counts were calculated from the anonymized and shuffled automatic judge outputs. Fractional wins were used to account for tied winners when multiple answers received the same highest overall score for a given question. In addition, a supplementary stratified blinded manual assessment was conducted on 30 benchmark questions, with five questions sampled from each of the six benchmark categories. For each sampled question, the three model responses were presented using anonymized answer labels and scored on a 1-to-5 scale. Mean manual scores and fractional win counts were calculated after mapping the anonymous labels back to model identities. This manual assessment was used as a complementary human check for the anonymized and shuffled 100-question automatic evaluation. The evaluation dimensions and normalized weights used for the final GCMembraneBench score are summarized in Table 2.

## 3. Results

### 3.1. Corpus and QA Dataset Outcomes

After the literature retrieval and two-stage screening, 582 papers were retained as the final literature corpus for GCMembrane-LLM. The corpus covered graphene membranes, CNT membranes, graphene/CNT hybrid membranes, filtration, desalination, water treatment, and related aqueous separation applications. This curated corpus provided the data foundation for full-text processing, QA generation, SFT, and RAG. The main corpus and QA dataset outcomes are summarized in Table 3.

Membrane-specific QA pairs were generated from the curated corpus through full-text preprocessing, Qwen-Max batch-task construction, batch inference, and response parsing. JSONL parsing, format conversion, preliminary rule-based cleaning, and deduplication produced 28,563 candidate QA pairs. A subsequent Qwen-3.5-plus-assisted quality-control step retained 12,208 QA pairs for the final SFT dataset, corresponding to a retention rate of 42.7%. Invalid records, duplicated questions, incomplete answers, overly short responses, malformed JSONL entries, weakly relevant QA pairs, and logically inconsistent QA pairs were removed during filtering. These procedures improved the alignment of the retained QA dataset with the intended application scope of GCMembrane-LLM.

The final QA dataset emphasized membrane mechanisms, nanochannel regulation, structure–performance relationships, practical limitations, and design strategies for filtration and desalination applications. The retained records also preserved distinctions among graphene membranes, GO laminates, CNT membranes, and graphene/CNT hybrid systems, which share related terminology but differ in transport pathways, structural regulation strategies, and separation mechanisms.

The manual spot-check of 100 randomly sampled retained QA pairs showed that 89 records were labeled KEEP and 11 records were labeled FLAG, corresponding to a KEEP rate of 89.0%. Within this sample, the pass rates for scientific plausibility and QA consistency were both 100.0%, while the pass rates for topic relevance and material–system distinction were both 89.0%. The flagged records were mainly associated with weak relevance or insufficient graphene/CNT membrane specificity, rather than obvious scientific errors or inconsistent QA pairs. This result indicates that the cleaned SFT dataset was generally suitable for domain-specific fine-tuning, while a small fraction of weakly related records may remain after automatic filtering.

### 3.2. Retrieval-Augmented Source Grounding

RAG was evaluated through representative source-grounded cases and a supplementary small-scale retrieval relevance assessment. The representative cases examined article-title and page-level source tracing, evidence-constrained answer generation, and membrane-specific evidence organization. Quantitative model comparison was conducted separately using GCMembraneBench under a direct-answer setting without retrieval context.

In the supplementary 30-query retrieval relevance evaluation, the RAG module retrieved the top four chunks for each membrane science query and each retrieved chunk was manually labeled for source relevance. The evaluation yielded a Hit@1 of 0.800, Hit@4 of 1.000, Precision@4 of 0.808, and MRR of 0.894. These results indicate that the retrieval module usually returned at least one relevant source within the top four retrieved chunks and frequently ranked a relevant source first. This analysis provides quantitative support for the source-retrieval component of the RAG workflow, although it evaluates retrieved source relevance rather than full sentence-level citation faithfulness.

**Figure 5:**
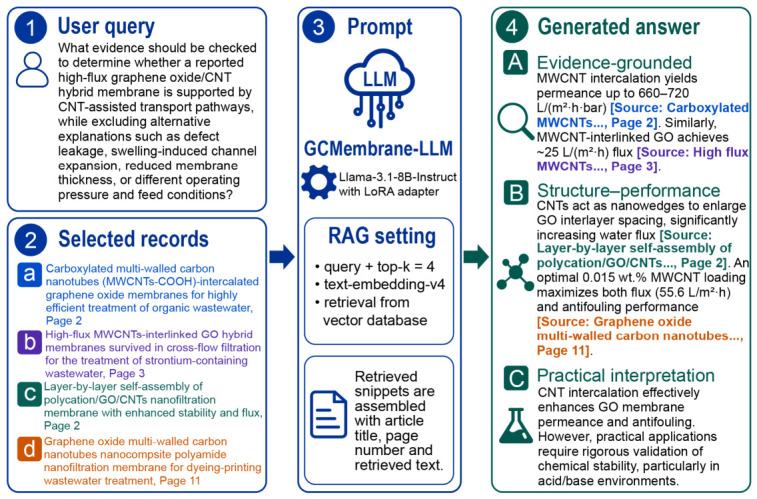
Representative retrieval-augmented source-grounded output for interpreting high-flux graphene oxide/carbon nanotube (GO/CNT) hybrid membrane performance. The example shows how GCMembrane-LLM responds to a membrane-specific question by using retrieved literature snippets with article-title and page-level metadata. For compact display, source-title strings in the generated-answer panel are abbreviated; the corresponding complete article titles are provided in the color-coded records a–d in the Selected records panel, and the cited page numbers identify the retrieved evidence pages. The query asks which evidence should be examined to determine whether high flux in a GO/CNT hybrid membrane is supported by CNT-assisted transport pathways, while also considering defect leakage, swelling-induced channel expansion, reduced membrane thickness, operating pressure, and feed conditions. The final output combines an evidence-supported answer with returned source information, enabling inspection of the literature basis of the response.

The source-grounding workflow improves response transparency and verifiability by returning page-level literature evidence together with the generated answer. Such traceability is important in membrane research because performance-related conclusions depend on material architecture, nanochannel structure, preparation method, feed composition, operating pressure, and testing conditions. By returning page-level source information together with the generated answer, the RAG module enabled the inspection of the literature basis of each response and helped to identify potential unsupported claims in source-grounded use cases [50,51]. Figure 5 presents a representative source-grounded output for evaluating a reported high-flux GO/CNT hybrid membrane. In this example, the retrieved snippets covered CNT-assisted transport pathways, GO/CNT interlayer regulation, water permeance, rejection or selectivity, antifouling behavior, acid/base stability, defect-related leakage, and operating condition effects. These snippets were assembled into a retrieval-augmented context to generate an evidence-supported answer with returned article-title and page-level source information.

### 3.3. Application-Oriented Membrane Structure–Performance Reasoning Case Studies

Three illustrative membrane science cases were selected to cover mechanistically distinct systems, namely GO/CNT composites, GO laminates, and CNT membranes. These cases were used to examine whether the RAG-enabled model could organize source evidence and connect membrane architecture, transport mechanisms, selectivity or rejection, swelling or defect risks, operating conditions, and practical interpretation; they were not treated as statistically sampled test cases or additional model-ranking experiments. The selected cases covered GO/CNT composite membranes, GO membranes, and CNT membranes, which are closely related in terminology but mechanistically distinct in graphene/CNT membrane research.

Case 1 examined GO/CNT composite membranes, with emphasis on the effects of CNT incorporation on water transport, selectivity, and stability. The case question was “How does CNT incorporation improve water transport in GO/CNT composite membranes, and under what conditions may it reduce selectivity or stability?” The selected evidence included CNT-intercalated GO membranes and related GO/CNT composite nanofiltration systems. CNT incorporation was linked to enlarged interlayer pathways, additional water-transport nanochannels, improved water permeance, enhanced antifouling behavior, and rejection performance. Potential limitations were also identified, including weak CNT–GO interactions, acid/base stability, CNT loading, defect formation, and long-term structural robustness. This case illustrates how GCMembrane-LLM organizes an evidence chain connecting CNT intercalation, GO lamellar architecture, transport enhancement, selectivity preservation, and stability constraints.

Case 2 examined GO membrane swelling and salt rejection, with emphasis on the effects of swelling, interlayer spacing, defects, and operating conditions on desalination performance. The case question was “How do swelling, interlayer spacing, defects, and operating conditions affect salt rejection in graphene oxide membranes for desalination?” The selected evidence covered external-pressure regulation of GO interlayer spacing, interlocked GO channels for forward-osmosis desalination, and molecular insights into multilayer GO desalination. Hydration-induced swelling was linked to enlarged interlayer spacing, which may increase water permeance but weaken ion sieving when salt species can access expanded GO galleries. Salt rejection was further associated with interlayer-spacing control, crosslinking or interlocking strategies, defect-related leakage, pressure conditions, and ion-transport pathways. This case illustrates how GCMembrane-LLM evaluates whether high permeance in GO membranes reflects controlled molecular sieving or is influenced by swelling, defects, or operating condition differences.

Case 3 examined high water flux and ion exclusion in CNT membranes. The case question was “In CNT membranes for water treatment or desalination, how do CNT pore diameter, inner-wall low-friction transport, hydration shell exclusion, entrance chemistry, membrane defects, and operating conditions jointly determine high water flux and ion exclusion?” The selected evidence covered CNT water permeation behavior, CNT membranes for water treatment applications, and zwitterion-CNT membranes with ion-responsive channels. Water-transport acceleration was distinguished from ion-exclusion requirements. CNT channels were associated with rapid water permeation through confined, low-resistance transport pathways, whereas ion exclusion was related to pore diameter, entrance chemistry, hydration-related barriers, channel functionalization, membrane defects, and operating conditions. Therefore, high water flux alone cannot sufficiently indicate selective desalination performance unless ion-rejection evidence and defect control are also verified.

The three cases collectively demonstrate that the RAG-enabled GCMembrane-LLM can organize source-grounded evidence chains across GO/CNT composite membranes, GO laminates, and CNT membranes. These case studies were used to illustrate evidence organization and membrane-specific structure–mechanism–condition reasoning, without being treated as an additional model-ranking experiment. The main outcomes of the three RAG-based membrane science cases are summarized in Table 4.

### 3.4. Comparative Performance on GCMembraneBench

**Figure 6:**
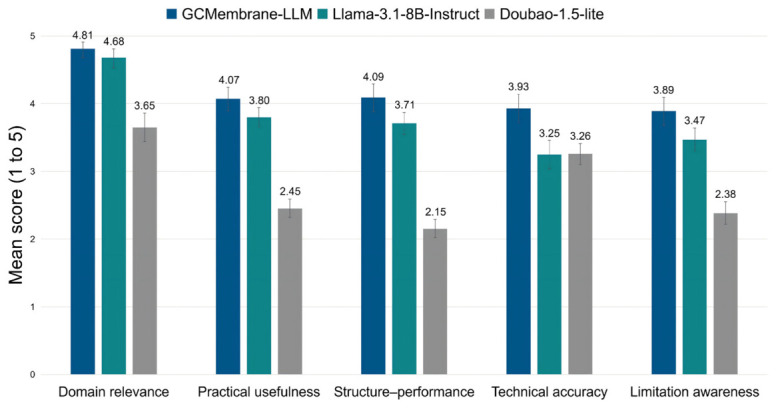
Dimension-level benchmark performance of GCMembrane-LLM and baseline models on GCMembraneBench under the anonymized and shuffled automatic evaluation protocol. Grouped bars show the mean scores of GCMembrane-LLM, Llama-3.1-8B-Instruct, and Doubao-1.5-lite across five evaluation dimensions: domain relevance, practical usefulness, structure–performance reasoning, technical accuracy, and practical limitation awareness. Error bars indicate bootstrap 95% confidence intervals estimated from question-level scores. GCMembrane-LLM achieved the highest mean score in all five dimensions, indicating stronger domain focus, application-oriented usefulness, structure–performance interpretation, technical accuracy, and awareness of practical membrane limitations.

Quantitative model comparison was performed using the 100-question GCMembraneBench under a direct-answer setting without retrieval context. This benchmark was used to assess the direct-answer performance associated with domain-specific SFT by comparing GCMembrane-LLM with two tested baseline models, Llama-3.1-8B-Instruct and Doubao-1.5-lite. The benchmark covered application-oriented graphene/CNT membrane questions related to GO membrane applications and mechanisms, CNT membrane transport and separation mechanisms, GO/CNT hybrid membrane design, cross-material structure–performance evaluation, application-condition and performance evaluation, and application-oriented design and troubleshooting. Model responses were evaluated across five domain-relevant dimensions: domain relevance, practical usefulness, structure–performance reasoning, technical accuracy, and practical limitation awareness. The dimension-level comparison is shown in Figure 6.

GCMembrane-LLM obtained the highest mean score among the three tested models across all five evaluation dimensions. For domain relevance, GCMembrane-LLM scored 4.81/5.0, compared with 4.68/5.0 for Llama-3.1-8B-Instruct and 3.65/5.0 for Doubao-1.5-lite. For practical usefulness, the corresponding scores were 4.07/5.0, 3.80/5.0, and 2.45/5.0, respectively. For structure–performance reasoning, GCMembrane-LLM achieved 4.09/5.0, compared with 3.71/5.0 for Llama-3.1-8B-Instruct and 2.15/5.0 for Doubao-1.5-lite. Higher mean scores were also obtained by GCMembrane-LLM in technical accuracy and practical limitation awareness, with values of 3.93/5.0 and 3.89/5.0, respectively.

Under the anonymized and shuffled five-dimension weighted scoring scheme, GCMembrane-LLM achieved a mean weighted score of 4.237/5.0, compared with 3.896/5.0 for Llama-3.1-8B-Instruct and 2.845/5.0 for Doubao-1.5-lite. Bootstrap analysis with 10,000 resampling iterations gave a 95% confidence interval of 4.087 to 4.378 for GCMembrane-LLM, 3.778 to 4.012 for Llama-3.1-8B-Instruct, and 2.723 to 2.968 for Doubao-1.5-lite. At the paired-question level, GCMembrane-LLM exceeded Llama-3.1-8B-Instruct by a mean paired difference of 0.341, with a 95% confidence interval of 0.143 to 0.533, and exceeded Doubao-1.5-lite by 1.392, with a 95% confidence interval of 1.177 to 1.596. The comparative performance summary is reported in Table 5.

The weighting sensitivity analysis showed that the model ranking remained unchanged under equal weighting and under alternative dimension-emphasis schemes that prioritized technical accuracy, practical usefulness, or structure–performance reasoning. This result indicates that the ranking was not dependent on a single weighting configuration. However, these results should be interpreted within the boundaries of the evaluation protocol. GCMembraneBench was an internal domain benchmark developed for graphene/CNT membrane question answering, and no retrieval context was provided during benchmark answer generation. Therefore, the score of 4.237/5.0 indicates improved direct-answer performance relative to the two tested baselines under the anonymized and shuffled automatic-judge protocol, rather than external validation on unseen membrane literature, comprehensive superiority over untested scientific or materials-domain LLM baselines, or quantitative evidence for the RAG component. These results suggest that domain-specific SFT improved direct-answer performance within the present internal benchmark setting, while source-grounded retrieval and evidence traceability were evaluated separately through the RAG results.

In addition to the anonymized and shuffled 100-question automatic evaluation, a supplementary stratified blinded manual assessment was further conducted on 30 benchmark questions, with five questions sampled from each of the six benchmark categories. For each question, the three model responses were presented using anonymized answer labels and scored on a 1-to-5 scale. GCMembrane-LLM achieved the highest mean manual score of 4.060/5.0, followed by Llama-3.1-8B-Instruct with 4.013/5.0 and Doubao-1.5-lite with 3.840/5.0. The corresponding fractional win counts were 14.33/30, 11.83/30, and 3.83/30, respectively. These results were consistent with the automatic evaluation ranking, although the manual score difference between GCMembrane-LLM and Llama-3.1-8B-Instruct was small. Therefore, the blinded manual assessment was interpreted as a supplementary human robustness check for the anonymized and shuffled automatic evaluation rather than a separate large-scale expert evaluation.

## 4. Discussion

### 4.1. From Literature Retrieval to Membrane Design Reasoning

GCMembrane-LLM is positioned as a source-grounded, domain-adapted reasoning interface for graphene/CNT membrane research [45,46]. Following the results presented above, its main value lies in supporting evidence-grounded interpretation, structure–mechanism–condition reasoning, and pre-experimental hypothesis organization. This role is particularly relevant for graphene/CNT membrane systems, where similar performance metrics may originate from different structural and experimental factors, including controlled nanochannel transport, enlarged interlayer spacing, defect-related leakage, reduced membrane thickness, feed composition, and operating pressure [3,4,5,6,7,17,18,19,20,21,22,23,24,32,33,34,35,36,37].

GCMembrane-LLM should therefore be interpreted within a defined application boundary. The framework is designed for evidence retrieval, evidence organization, cross-study comparison, and preliminary hypothesis formulation before experimental validation. Autonomous membrane design, direct performance prediction, and the replacement of transport experiments remain outside its intended scope. Therefore, model outputs should be used to examine whether a reported membrane performance claim is supported by consistent structure–mechanism–condition evidence, especially when flux or permeance, selectivity or rejection, fouling resistance, swelling stability, defect control, operating conditions, and durability are coupled.

The representative membrane science cases clarify this evidence-organization role across three mechanistically distinct membrane architectures. For GO/CNT composite membranes, CNT-enabled transport pathways can be examined together with selectivity loss, CNT loading, dispersion, interfacial compatibility, and defect formation. For GO laminates, high permeance can be interpreted together with swelling, interlayer-spacing variation, crosslinking or interlocking strategies, and salt rejection evidence. For CNT membranes, low-resistance water transport can be distinguished from ion-exclusion requirements related to pore diameter, entrance chemistry, hydration barriers, channel functionalization, membrane defects, and operating conditions. These examples show how the framework supports evidence chain construction across related carbon-based membrane architectures with distinct transport pathways and separation mechanisms [21,22,23,24,32,33,34,35,36,37,38].

The comparative benchmark results further suggest that membrane-specific SFT improved the ability of the general instruction model to generate application-oriented graphene/CNT membrane responses under direct-answer evaluation without retrieval context. Higher scores in domain relevance, practical usefulness, structure–performance reasoning, technical accuracy, and practical limitation awareness indicate stronger alignment with membrane science questions than the two baseline models. The RAG-based examples provide a separate demonstration of source-grounded traceability and qualitative evidence chain construction. Together, these results support the use of GCMembrane-LLM as an evidence-assisted reasoning interface for literature-based interpretation and preliminary hypothesis organization, while experimental verification remains necessary for any membrane design or performance claim.

### 4.2. Source Grounding, Reproducibility, and Scientific Usability

A key feature of the proposed framework is the integration of RAG for source-grounded inference [50]. In scientific applications, unsupported generation is particularly problematic because membrane performance claims are strongly affected by material composition, nanochannel structure, preparation method, feed composition, operating pressure, and testing duration. The relevant literature chunks are retrieved before answer generation, and article-title and page-level source information is returned with the generated response, thereby improving response transparency and verifiability, as illustrated in Figure 4 and Figure 5 [50,51].

The RAG-enabled source-grounding design further improves the scientific usability of GCMembrane-LLM. Compared with a standalone fine-tuned model, the RAG-enabled configuration provides a route for inspecting the literature basis of generated answers. This function is particularly important in graphene/CNT membrane research, where similar terminology may refer to distinct material systems, including nanoporous graphene membranes, GO laminates, CNT membranes, and graphene/CNT hybrid membranes [21,22,23,24,32,33,34]. Because source metadata are retained during retrieval and answer generation, the returned evidence can be checked against the membrane architecture and separation context of the query.

Source grounding should therefore be interpreted with methodological caution, because it does not guarantee factual completeness. The quality of a RAG-based answer remains dependent on corpus coverage, text-extraction quality, chunking strategy, embedding performance, retrieval specificity, and prompt design [50,51]. Retrieved evidence may be incomplete, overly broad, or only partially aligned with the query. GCMembrane-LLM should therefore be used as an evidence-assisted reasoning tool, with outputs requiring expert interpretation when supporting literature screening, performance comparison, or preliminary experimental planning.

### 4.3. Limitations, Evaluation Boundaries, and Future Directions

The current framework has limitations related to corpus coverage, corpus representation, evidence-type labeling, benchmark design, RAG evaluation, and transferability. First, the performance of GCMembrane-LLM remains dependent on the coverage and balance of the curated literature corpus. Although the corpus was constructed to cover graphene/CNT membranes for filtration, desalination, water treatment, and related aqueous separations, certain membrane systems, feed compositions, operating conditions, long-term stability tests, and performance metrics may still be underrepresented. Rule-based QA topic analysis also showed that fouling/antifouling, swelling/stability, and scale-up or practical-limit topics were less frequent than structure, transport, performance, and operating condition topics in the final SFT dataset. Future dataset expansion should further strengthen these underrepresented application limitation categories.

Second, the present corpus representation remained primarily text based. The workflow retained article titles, PDF filenames, page numbers, source paths, and extracted text for traceability, but it did not structurally parse numerical tables, plotted data, image panels, or supplementary datasets. This limitation is important for membrane science because the key evidence for permeability, rejection, fouling resistance, operating pressure, feed concentration, membrane thickness, testing duration, and long-term stability is often reported in tables, figures, and supplementary files. Therefore, source-grounded answers from the current RAG module are more reliable for qualitative mechanism organization than for complete numerical performance extraction across studies. When numerical comparison is required, the returned page-level sources should be inspected manually against the original tables, figures, and experimental conditions.

Third, GO and CNT membrane studies include both molecular simulation reports and experimental membrane-fabrication studies, and these evidence types support different levels of inference. Simulation studies are useful for interpreting confined transport, hydration barriers, pore-size effects, and ion exclusion mechanisms, whereas experimental studies are required to verify membrane fabrication, defect control, fouling behavior, pressure-dependent performance, and long-term stability. In the current workflow, explicit evidence-type labels, such as simulation, experiment, review, or mixed study, were not used as primary training fields. Future corpus construction should add evidence-type labeling so that simulation-derived inference and experimental validation can be handled more transparently during retrieval and answer generation.

Fourth, the benchmark results should be interpreted within the boundaries of the evaluation protocol. GCMembraneBench was developed as an internal domain benchmark for graphene/CNT membrane question answering, and no retrieval context was provided during benchmark answer generation. Question-level textual isolation was re-checked against all 12,208 retained SFT QA records, showing no exact duplicates, no very high textual overlaps, two partial textual overlaps, and 98 questions with no direct textual duplication. However, because the benchmark file did not contain source paper identifiers or DOI fields, paper-level source isolation and semantic leakage could not be assessed. In addition, the model comparison was limited to the original Llama-3.1-8B-Instruct base model and one general online model endpoint. Stronger scientific, chemistry, materials-domain, or membrane-engineering LLM baselines were not included, and the results should therefore be interpreted as evidence of improvement over the two tested baselines without extending the claim to untested scientific or materials-domain LLM baselines. To reduce identity and position bias in the automatic judge, the full 100-question comparison was rerun using anonymized answer labels and shuffled answer order. This revised automatic evaluation gave the same model ranking, with GCMembrane-LLM achieving a mean weighted score of 4.237/5.0 and 62.5 fractional wins out of 100. Nevertheless, the evaluation still relied on a single LLM-as-a-judge protocol, and the supplementary blinded manual assessment remained limited to 30 questions without multiple independent raters or inter-rater agreement analysis. Future evaluation should include stronger scientific and materials-domain baselines, larger anonymized human assessment, multiple membrane experts, inter-rater agreement analysis, paper-level source isolation, and external validation using unseen literature.

Fifth, the RAG evaluation was strengthened by a supplementary small-scale retrieval relevance assessment. Thirty membrane science queries were used to examine whether the top four retrieved chunks contained source evidence relevant to the query. The retrieval module achieved a Hit@1 of 0.800, Hit@4 of 1.000, Precision@4 of 0.808, and MRR of 0.894, indicating that relevant evidence was usually retrieved within the top four results and was often ranked first. However, this analysis focused on retrieved source relevance at the chunk level. A full sentence-level citation faithfulness evaluation, including systematic citation accuracy, source support rate, unsupported claim rate, and hallucination reduction analysis, was not conducted. Future work should therefore include larger retrieval-grounding benchmarks with independent source inspection and claim-level evidence verification.

Finally, the workflow can be extended to other membrane material families, but direct transfer requires material-specific corpus reconstruction and quality control. For polymeric, ceramic, MXene, covalent organic framework, metal–organic framework, or biomimetic membranes, the literature search terms, exclusion rules, QA-generation prompts, evaluation dimensions, and RAG metadata should be redesigned to reflect the corresponding transport mechanisms, stability issues, operating windows, and application constraints. Thus, the present framework provides a transferable development route, including corpus curation, QA generation, SFT, retrieval indexing, and source-grounded evaluation, while the trained GCMembrane-LLM itself should be regarded as specific to graphene/CNT membrane literature unless further adapted with material-specific data.

Overall, GCMembrane-LLM highlights the potential of domain-specific and source-grounded language models for organizing graphene/CNT membrane literature and supporting application-oriented membrane reasoning. Its main contribution lies in evidence retrieval, evidence organization, and the preliminary interpretation of relationships among membrane architecture, nanoconfined transport, selectivity, fouling resistance, swelling stability, defect control, durability, operating conditions, and practical limitations. As a literature-grounded reasoning interface, GCMembrane-LLM supports evidence-based knowledge interpretation and pre-experimental hypothesis organization, while experimental validation remains necessary for membrane design or performance claims.

## 5. Conclusions

GCMembrane-LLM was developed as an evidence-grounded, domain-specific LLM for graphene and carbon nanotube membrane research. Using a curated corpus of 582 papers, 12,208 cleaned membrane-specific QA pairs were generated and used for LoRA-based supervised fine-tuning of Llama-3.1-8B-Instruct, with RAG further integrated to provide article-title and page-level source traceability. A supplementary 30-query retrieval relevance evaluation further showed a Hit@1 of 0.800, Hit@4 of 1.000, Precision@4 of 0.808, and MRR of 0.894 for the top four retrieved chunks. On the 100-question GCMembraneBench under a direct-answer setting without retrieval context, anonymized and shuffled automatic evaluation showed that GCMembrane-LLM achieved the highest mean weighted score of 4.237/5.0, and a supplementary stratified 30-question blinded manual assessment showed the same ranking. Representative RAG-based cases showed that CNT-assisted GO/CNT transport should be interpreted together with dispersion, interfacial compatibility, defects, and stability; GO desalination requires attention to swelling control, interlayer spacing, and defect suppression; and CNT-enabled high flux must be evaluated with pore diameter, entrance chemistry, hydration barriers, ion rejection, and operating conditions. These results indicate that GCMembrane-LLM can support source-traceable evidence organization, structure–mechanism–condition interpretation, and preliminary hypothesis formulation before experimental validation. The current framework remains limited by its internal benchmark setting, limited baseline scope, the use of a single automatic judge, the absence of sentence-level citation faithfulness evaluation, text-based corpus representation, limited independent human assessment, and the need to distinguish simulation-derived evidence from experimental membrane validation in future versions.

## Figures and Tables

**Table 1:** Core supervised fine-tuning configuration of GCMembrane-LLM.

Parameter	Setting
Base model	Llama-3.1-8B-Instruct
Fine-tuning framework	LLaMA-Factory
Training stage	Supervised fine-tuning
Fine-tuning method	LoRA
SFT dataset size	12,208 records
Dataset format	instruction, input, output
LoRA rank	r = 8
LoRA scaling factor	α = 16
LoRA dropout	0.0
LoRA target modules	q_proj, k_proj, v_proj, o_proj, gate_proj, up_proj, down_proj
Maximum sequence length	2048 tokens
Number of training epochs	3
Per-device training batch size	2
Gradient accumulation steps	4
Effective batch size	8
Learning rate	5 × 10^−5^
Learning-rate scheduler	Cosine scheduler
Warm-up ratio	0.1
Precision	FP16
Optimizer	Paged AdamW 8-bit
Flash attention	Auto
Gradient checkpointing	Enabled

**Table 2:** Evaluation dimensions and normalized weights used for the final GCMembraneBench score.

Symbol	Evaluation Dimension	Weight
D	Domain relevance	0.278
A	Practical usefulness	0.222
S	Structure–performance reasoning	0.222
T	Technical accuracy	0.167
L	Practical limitation awareness	0.111

**Table 3:** Summary of literature corpus and QA dataset outcomes.

Item	Result
Final literature corpus	582 papers
Candidate QA pairs generated	28,563
Final cleaned QA pairs retained	12,208
QA retention rate	42.7%

**Table 4:** Representative RAG-based membrane science cases evaluated using GCMembrane-LLM.

Case	System and Reasoning Target	Main Finding
Case 1	GO/CNT composites; CNT incorporation, transport, selectivity, and stability	CNTs can enlarge GO transport pathways and improve permeance, but CNT loading, dispersion, interfacial compatibility, and defects must be checked.
Case 2	GO laminates; swelling, interlayer spacing, and salt rejection	GO swelling can enlarge interlayer spacing and weaken ion sieving; spacing control and defect suppression are critical for salt rejection.
Case 3	CNT membranes; high flux, pore diameter, and ion exclusion	CNT channels can accelerate water transport, but ion exclusion still depends on pore size, entrance chemistry, hydration barriers, defects, and operating conditions.

**Table 5:** Comparative performance summary from the anonymized and shuffled 100-question GCMembraneBench automatic evaluation.

Model	Score	95% Confidence Interval (CI)	Δ Score	Δ 95% CI	Fractional Wins
GCMembrane-LLM	4.237	4.087 to 4.378	NA	NA	62.5
Llama-3.1-8B-Instruct	3.896	3.778 to 4.012	0.341	0.143 to 0.533	33.5
Doubao-1.5-lite	2.845	2.723 to 2.968	1.392	1.177 to 1.596	4.0

Δ score was calculated as the GCMembrane-LLM score minus the corresponding baseline-model score for the same benchmark question. Fractional wins account for tied winners among model responses.

## Data Availability

The data supporting the findings of this study are provided in the Supplementary Materials, including the benchmark dataset, anonymized and shuffled benchmark answer table, private answer-label mapping record, model output files, judge score tables, confidence interval summaries, paired differences results, train–test textual isolation table, supplementary blinded manual scoring table, small-scale RAG retrieval relevance records, configuration files, and key processing scripts. Private credentials, including API keys, access tokens, endpoint secrets, OSS access information, and account-specific authentication information, are not required for understanding the reported workflow and should be configured by users in their own computing environments when reproducing API-dependent steps. The original full-text PDF corpus is not redistributed due to publisher copyright restrictions.

## Supplementary Materials

The following supporting information can be downloaded at: https://www.mdpi.com/article/10.3390/membranes16060214/s1. The supporting information includes literature retrieval terms, metadata schemas, QA-generation and cleaning records, SFT and RAG configuration files, benchmark questions, anonymized and shuffled benchmark answer files, private answer-label mapping records, model outputs, judge score tables, confidence interval summaries, paired differences results, weighting sensitivity analysis, train–test textual isolation results, supplementary blinded manual scoring records, small-scale RAG retrieval relevance records, representative RAG case records, and the processing scripts used in this study.

## Abbreviations

The following abbreviations are used in this manuscript:

AI	Artificial Intelligence
API	Application Programming Interface
CI	Confidence Interval
CNT	Carbon Nanotube
CSV	Comma-Separated Values
DOI	Digital Object Identifier
DSW	Data Science Workshop
FP16	16-Bit Floating-Point Precision
GO	Graphene Oxide
JSON	JavaScript Object Notation
JSONL	JavaScript Object Notation Lines
LLM	Large Language Model
LoRA	Low-Rank Adaptation
OSS	Object Storage Service
PDF	Portable Document Format
QA	Question–Answer
RAG	Retrieval-Augmented Generation
SFT	Supervised Fine-Tuning
TF-IDF	Term Frequency–Inverse Document Frequency
