# Supplementary material for: GCMembrane-LLM: An Evidence-Grounded Domain-Specific Large Language Model for Structure–Performance Reasoning in Graphene and Carbon Nanotube Separation Membranes
Source: Membranes (Basel). 2026 Jun 21;16(6):214. doi: 10.3390/membranes16060214 (PMC13304321; doi:10.3390/membranes16060214)
Supplement: Supplementary file 1 [file membranes-16-00214-s001.zip › membranes-4369928-supplementary.pdf]

Supplementary Materials

These Supplementary Materials provide detailed methodological materials to support the reproducibility and inspectability of GCMembrane-LLM, including literature retrieval, corpus screening, PDF processing, metadata preservation, QA generation, QA cleaning, LoRA supervised fine-tuning, retrieval-augmented generation, small-scale RAG retrieval relevance evaluation, benchmark construction, train–test textual isolation checking, anonymized and shuffled automatic judging, model comparison, supplementary blinded manual assessment, bootstrap confidence interval analysis, weighting sensitivity analysis, and representative RAG case study outputs. The complete 100-question benchmark dataset, anonymized and shuffled benchmark-answer table, private answer-label mapping record, model responses from GCMembrane-LLM, Llama-3.1-8B-Instruct, and Doubao-1.5-lite, Qwen judge-score logs, confidence-interval summaries, paired bootstrap difference results, train-test textual isolation records, blinded manual scoring records, small-scale RAG retrieval relevance records, and key processing scripts are provided as separate machine-readable supplementary files in .jsonl, .csv, .xlsx, .yaml, .txt, and .py formats.

Security Note: Private credentials, including API keys, access tokens, endpoint secrets, OSS access information, and account-specific authentication details, are not required for understanding the reported workflow. Users should configure these credentials in their own computing environments when reproducing API-dependent steps.

Table S1. Full OpenAlex query terms and exclusion terms used for literature retrieval.

Category	Setting or term list
Database	OpenAlex Works API.
Record type and DOI requirement	Type: article; has_doi: true.
Publication year filter used in uploaded retrieval code	publication_year: >=2014 and publication_year: <2027. This corresponds to records from 2014 through 2026.
Ranking criterion	cited_by_count:desc.
Maximum retrieval limit in uploaded code	800 records before deduplication.
Core material terms	"Graphene–carbon nanotube composite membrane"; "graphene/CNT composite membrane"; "GO-CNT composite membrane"; "graphene oxide–carbon nanotube hybrid membrane"; "graphene–CNT hybrid membrane"; "GO/CNT membrane"; "CNT–graphene membrane"; "CNT-GO membrane"; "graphene membrane"; "carbon nanotube membrane"; "graphene oxide membrane"; "GO membrane".
Application terms	Desalination; water desalination; filtration; nanofiltration; ultrafiltration; reverse osmosis; forward osmosis; water purification; water treatment; wastewater treatment; ion separation; salt rejection; molecular sieving; water permeation; membrane separation.
Exclusion terms	Photocatalysis; photocatalytic; hydrogen; battery; supercapacitor; sensor; electrode; CO2 electrolysis; terahertz; therapy; cancer; imaging; solar cell; alloy; microplastic; MXene; MoS2; MoS2 variant spellings; molybdenum disulfide; actuator; robot; ceramic; foam; triboelectric; piezoresistive.
Fields saved in retrieval	Title; DOI; year; citations; Is_Open_Access; journal; abstract.

Category	Setting or term list
Output	
Abstract handling	OpenAlex abstract_inverted_index was reconstructed into a readable abstract by sorting indexed word positions.

Code S1. Python script template for Openalex-based literature retrieval and metadata collection.

literature retrieval.py

Table S2. Metadata fields retained for full-text processing and source traceability.

Metadata field	Description	Use in the workflow
paper_id	Unique identifier assigned to each paper in the curated corpus.	Used for document tracking, chunk indexing, and linking extracted text to the original paper.
article_title	Full article title associated with the source PDF.	Used for source display in retrieval-augmented generation and for tracing generated answers back to the literature.
pdf_name	Original PDF filename of the literature document.	Used for file matching, document management, and verification of the source document.
page_number	Page number from which the text was extracted.	Used for page-level source tracing and evidence verification during RAG-based question answering.
oss_path	Storage path of the PDF file in Alibaba Cloud Object Storage Service.	Used to locate the original document and support reproducibility of corpus processing.
text	Extracted and cleaned text from the corresponding page or text chunk.	Used as input for question–answer generation, vector indexing, retrieval, and answer generation.

Code S2. PDF text preprocessing and Qwen-Max batch-task construction for membrane-specific QA generation.

section 2.2.py

Table S3. Workflow for membrane-specific QA generation and data cleaning.

Stage	Step	Operation	Output
QA generation	Corpus-to-task preparation	Convert cleaned full-text corpus into Alibaba Cloud Model Studio JSONL batch-task files	JSONL batch-task files
QA generation	Batch inference	Submit JSONL batch-task files to Qwen-Max through Alibaba Cloud Model Studio	Raw nested JSONL response files
QA generation	QA extraction	Extract model-generated QA pairs from batch response contents	Raw membrane-specific QA pairs

Data cleaning	Format conversion	Convert extracted QA pairs into LLaMA-Factory instruction/input/output format	Initial SFT-format QA dataset
Data cleaning	Rule-based cleaning	Remove empty records, malformed outputs, duplicated questions, and overly short answers	28,563 candidate QA pairs
Data cleaning	LLM-based quality control	Use Qwen-3.5-plus to judge KEEP or DISCARD	12,208 retained QA pairs

Code S3. Python script for parsing Qwen-Max batch outputs and constructing LLaMA-Factory SFT-format QA data.

clean_jsonl(1)(1).py

Code S4. Python script for Qwen-3.5-plus-based KEEP/DISCARD quality-control filtering.

agent_filter(1).py

Table S4. Dataset-count summary before and after QA data cleaning.

Dataset stage	Description	Number of QA pairs
Candidate QA dataset	Membrane-specific QA pairs after JSONL parsing, format conversion, and preliminary rule-based cleaning	28,563
Removed QA pairs	Invalid, duplicated, incomplete, overly short, weakly relevant, or logically inconsistent QA pairs	16,355
Final cleaned QA dataset	QA pairs retained for supervised fine-tuning after Qwen-3.5-plus KEEP/DISCARD quality control	12,208
Retention rate	12,208 / 28,563	42.74%

Table S4a. Topic distribution of the final cleaned QA dataset used for supervised fine-tuning.

QA type	Number	Proportion
membrane structure	3954	32.39%
transport mechanism	2459	20.14%
separation performance	2489	20.39%
fouling/antifouling	285	2.33%
swelling/stability	985	8.07%
operating conditions	4188	34.31%
scale-up/practical limits	795	6.51%

The QA-topic distribution was obtained by rule-based multi-label keyword matching over the instruction, input, and output fields of the final cleaned SFT dataset. Because one QA record may involve more than one topic, the proportions are not mutually exclusive.

Table S4b. Manual spot-check protocol and summary for 100 sampled QA pairs.

Item	Setting or result
Source dataset	Final cleaned SFT dataset
Total SFT records	12,208
Sampling method	Random sampling
Random seed	20260526
Number of inspected QA pairs	100
Inspection criteria	Topic relevance to graphene/CNT membrane separations; scientific plausibility; question-answer consistency; material-system distinction
Decision labels	KEEP or FLAG
KEEP records	89
FLAG records	11
KEEP rate	89.0%
Topic relevance pass rate	89.0%
Scientific plausibility pass rate	100.0%
QA consistency pass rate	100.0%
Material-system distinction pass rate	89.0%
Main flagged issue	Weak relevance or insufficient graphene/CNT membrane specificity

File S4b. Manual spot-check records for 100 sampled QA pairs.

100 QA manual spot check(1).csv.

Table S5. Full supervised fine-tuning configuration of GCMembrane-LLM.

Parameter	Setting
Model path	/mnt/workspace/models/Llama-3.1-8B-Instruct
Training stage	sft
Training mode	do_train: true
Fine-tuning type	lora
LoRA target	all
Dataset directory	/mnt/workspace/LLaMA-Factory/data
Dataset alias	my_physics_train
Dataset file	hegeshuju.jsonl
Dataset records	12,208
Dataset fields	instruction, input, output
Training template	Llama3
Cutoff length	2048
Learning rate	0.00005

Epochs	3.0
Maximum samples	100000
Per-device training batch size	2
Gradient accumulation steps	4
Learning-rate scheduler	cosine
Logging steps	5
Save steps	500
Warm-up ratio	0.1
Output directory	/mnt/workspace/LLaMA-Factory/saves/Llama-3.1-8B-Instruct/lora/sft
BF16	false
FP16	true
Flash attention	auto
Optimizer	paged_adamw_8bit
Gradient checkpointing	true

The training template records the setting used in the submitted LLaMA-Factory training configuration, whereas inference was performed using the deployed Llama-compatible serving configuration described in the corresponding inference scripts.

File S1. LLaMA-Factory training YAML configuration.

Code_S5_1_training_yaml.yaml

File S2. LLaMA-Factory dataset registration entry.

Code_S5_2_dataset_registration.json

File S3. PEFT LoRA adapter configuration.

adapter_config.json

Code S5. JSONL format validation script.

Code_S5_4_validate_sft_jsonl.py

Code S5a. Python script for rule-based QA-topic distribution analysis.

Code_S5a_QA_topic_distribution.py

File S4. Final supervised fine-tuning dataset.

Supplementary_Data_S2_final_SFT_dataset.jsonl

File S4a. QA-topic distribution of the final cleaned SFT dataset.

qa_topic_distribution.csv

Table S6. Metadata fields retained in the RAG workflow.

Field name	Description	Used in
paper_id	Unique identifier assigned to each paper	Metadata table, chunk construction, retrieval records
article_title	Full article title	Source-grounded context and final source display
pdf_name	PDF filename	File tracing and reproducibility
oss_path	Alibaba Cloud OSS storage path	Corpus storage tracing
page_number	Page number recorded during PDF text extraction	Page-level evidence tracing
chunk_id	Unique identifier assigned to each text chunk	Vector indexing and retrieval
text	Page-level chunk text	Embedding, retrieval, and source-grounded context construction
rank	Retrieval rank of each returned chunk	Retrieval inspection
distance	vector distance returned by the ChromaDB similarity search	Retrieval inspection
query	User query used for retrieval	Retrieval logging

Table S6a. Scope and limitations of RAG source-grounding evaluation after supplementary retrieval relevance assessment.

Metric	Definition	Result
top 4 retrieval relevance	Whether the retrieved top 4 records are semantically related to the input membrane science query.	Quantified using a supplementary 30-query retrieval relevance evaluation. The RAG module achieved Hit@1 = 0.800, Hit@4 = 1.000, Precision@4 = 0.808, and MRR = 0.894.
Source support rate	Whether the generated answer sections are supported by returned article-title and page-level evidence.	Demonstrated through source-grounded answer examples with article-title and page-level source tracing. A full sentence-level source support audit was not systematically conducted.
unsupported claim rate	Whether generated statements lack support from the retrieved snippets.	Not systematically measured in this study.

This study used RAG mainly to provide article-title and page-level source tracing for membrane science answers. The supplementary 30-query evaluation quantified retrieval relevance for the top four retrieved chunks, while full sentence-level citation faithfulness, citation accuracy, source support rate, unsupported claim rate, and hallucination reduction remain future evaluation directions.

Table S6b. Small-scale RAG retrieval relevance evaluation protocol.

Item	Setting
Number of evaluation queries	30
Query categories	GO swelling and salt rejection; CNT transport and ion exclusion; GO/CNT hybrid

Item	Setting
	transport; fouling and antifouling; operating conditions; defects and practical limitations
Questions per category	5
Retrieval setting	Top k = 4 retrieved chunks per query
Retrieved fields exported for inspection	query_id; category; question; rank; distance; paper_id; article_title; page_number; pdf_name; oss_path; retrieved_text
Manual relevance label	is_relevant_gold = 1 for relevant retrieved evidence; is_relevant_gold = 0 for irrelevant retrieved evidence
Support-level labels	strong; partial; irrelevant
Metrics calculated	Hit@1; Hit@4; Precision@4; mean reciprocal rank (MRR)
Evaluation boundary	Retrieved-source relevance was evaluated at the chunk level. Sentence-level citation faithfulness was not systematically audited.

Table S6c. Small-scale RAG retrieval relevance evaluation results.

Metric	Value	Interpretation
Hit@1	0.800	In 80.0% of the 30 queries, the first retrieved chunk was manually labeled as relevant.
Hit@4	1.000	Every query had at least one relevant chunk within the top four retrieved results.
Precision@4	0.808	On average, 80.8% of the top four retrieved chunks were labeled as relevant.
MRR	0.894	Relevant evidence was usually ranked near the top of the retrieved list.

Code S6. Paper-level metadata reconstruction for RAG indexing.

Code_S6_1a_metadata_rebuild.py

Code S7. Page-level PDF text extraction, chunking, and metadata preservation.

Code_S6_1b_page_chunking.py

Code S8. Embedding generation and ChromaDB vector database indexing.

Code_S6_2_embedding_chromadb_indexing.py

Code S9. Query retrieval, source-grounded context construction, and prompt-based answer generation.

Code_S6_3_retrieval_prompt_generation.py

File S6b. Small-scale RAG retrieval relevance evaluation workbook.

rag_eval_gold_labels_filled_expert.xlsx

File S6c. RAG retrieval records for the 30-query retrieval relevance evaluation.

rag_eval_retrieval_records.csv

File S6d. RAG evaluation query set.

rag_eval_questions_30.csv

Code S9a. Python script for batch RAG retrieval relevance evaluation.

batch_rag_grounding_eval.py

Code S9b. Python script for calculating RAG retrieval relevance metrics.

calculate_rag_retrieval_metrics.py

Code S10. Lightweight Flask demonstration and retrieval logging.

Code_S6_4_flask_demo.py

Table S7. Category distribution and research purposes of the 100-question GCMembraneBench benchmark.

**Category**	**Number of questions**	**Purpose**
GO membrane applications and mechanisms	20	Interlayer spacing, swelling control, water flux, salt rejection, selectivity, stability, antifouling, and water-treatment applications.
CNT membrane transport and separation mechanisms	20	Low-friction water transport, CNT pore diameter, channel alignment, functionalization, ion exclusion, defects, fouling, and fabrication challenges.
GO/CNT hybrid membrane design	20	CNT spacer effects, CNT loading, dispersion, channel continuity, flux enhancement, swelling suppression, defect control, and hybrid membrane scalability.
Cross-material structure–performance evaluation	15	Cross-material reasoning linking pore size, interlayer spacing, surface charge, hydrophilicity, defects, thickness, stability, and testing conditions with membrane performance.
Application-condition and performance evaluation	15	Performance metrics, flux-rejection balance, fouling tests, stability tests, feed concentration, pressure, pH, multi-contaminant water, cost, scalability, and environmental safety.
Application-oriented design and troubleshooting	10	Application-oriented troubleshooting for GO, CNT, and hybrid membranes, including low flux, poor rejection, fouling, swelling, scale-up, and future application directions.

The six benchmark categories contain 100 questions in total.

Table S8. Unified output schema for model benchmark responses

Field	Description
id	Question ID in GCMembraneBench
category	Main benchmark category
subtopic	Specific membrane-related subtopic
difficulty	Question difficulty label
status	Question status
question	Benchmark question
model_name	Evaluated model name
answer	Model-generated answer
run_status	Running status of the inference call
elapsed_seconds	Response time for each question

Table S9. Inference settings used for model comparison.

Model	Inference mode	Running environment	Model setting
GCMembrane-LLM	Local inference	Development instance	Llama-3.1-8B-Instruct loaded with the trained LoRA adapter
Llama-3.1-8B-Instruct	Local inference	Same development instance	Original base checkpoint without LoRA adapter
Doubao-1.5-lite	Online endpoint inference	Volcengine Ark endpoint	Endpoint-based model access

Table S10. Scoring rubric and normalized weights used by Qwen-3.5-plus

Symbol	Dimension	Score range	Weight	Used in final score	Evaluation focus
D	Domain relevance	1–5	0.278	Yes	Relevance to graphene, CNT, and graphene/CNT membrane research
A	Practical usefulness	1–5	0.222	Yes	Usefulness for filtration, desalination, wastewater treatment, water purification, and membrane design
S	Structure–performance reasoning	1–5	0.222	Yes	Ability to connect membrane structure with separation performance
T	Technical accuracy	1–5	0.167	Yes	Scientific correctness and absence of misleading mechanisms
L	Practical limitation awareness	1–5	0.111	Yes	Awareness of swelling, fouling, stability, cost, scale-up, and operational limits
C	Clarity/conciseness	1–5	Auxiliary	No	Clarity, organization, and appropriate answer length

The clarity/conciseness dimension was retained as an auxiliary diagnostic indicator and was excluded from the final weighted overall-score calculation.

Table S10a. Automatic judge transparency settings used for GCMembraneBench evaluation.

Item	Setting
Judge model	Qwen-3.5-plus
API interface	Alibaba Cloud DashScope compatible-mode API
Temperature	0
Maximum output tokens	1600
Input to judge	Benchmark question and three anonymized model-generated answers
Compared models	GCMembrane-LLM, Llama-3.1-8B-Instruct, and Doubao-1.5-lite
Model identity handling	Model identifiers were removed from the judge prompt and replaced by anonymized answer labels.
Answer order	For each benchmark question, the three model responses were randomly assigned to Answer A, Answer B, and Answer C.
Private mapping	The private answer-label-to-model mapping was used only after scoring to aggregate model-level results.
Scoring scale	1 to 5
Scored dimensions	domain relevance, practical usefulness, structure–performance reasoning, technical accuracy, practical limitation awareness, and clarity/conciseness
Raw judge output	JSON-formatted dimension scores, brief reasons, and anonymized winner label
Final reported score	Recalculated from five domain-relevant dimensions after excluding clarity/conciseness
Human re-scoring	Supplementary stratified blinded manual assessment was conducted on 30 benchmark questions as a complementary human check.
Limitation	The evaluation still relied on a single LLM-as-a-judge protocol, and the 30-question manual assessment did not include multiple independent raters or inter-rater agreement analysis.

Table S10b. Score-level interpretation used for automatic judging.

Score	Interpretation
1	Incorrect, irrelevant, misleading, or unrelated to graphene/CNT membrane research.
2	Partly relevant but generic, shallow, or missing important membrane-specific information.
3	Generally relevant and mostly correct, but limited in structure–performance reasoning, application usefulness, or practical interpretation.
4	Scientifically reasonable, domain-relevant, and useful, with clear graphene/CNT membrane reasoning and only minor omissions.
5	Highly accurate, domain-specific, application-oriented, and clearly connects membrane structure, transport mechanism, separation performance, and practical limitations.

Table S10c. Automatic judge dimensions and scoring focus.

Dimension in judge script	Corresponding manuscript dimension	Scoring focus
domain_relevance	Domain relevance	Focus on graphene membranes, CNT membranes, and graphene/CNT hybrid membranes.
application_usefulness	Practical usefulness	Usefulness for filtration, desalination, wastewater treatment, water purification, and practical membrane design.
structure_performance_logic	Structure–performance reasoning	Ability to connect interlayer spacing, pore size, CNT loading, dispersion, surface chemistry, defects, thickness, or support layers with membrane performance.
technical_accuracy	Technical accuracy	Scientific correctness and avoidance of misleading mechanisms or unsupported absolute claims.
practical_limitations	Practical limitation awareness	Awareness of swelling, fouling, mechanical stability, chemical stability, defect control, long-term operation, cost, and scale-up.
clarity_conciseness	Auxiliary clarity/conciseness	Clarity, organization, and appropriate answer length. This dimension was excluded from the final reported score.

The automatic judge prompt requested Qwen-3.5-plus to evaluate the three anonymized model answers independently for each benchmark question and to return a structured JSON object containing six dimension scores, brief reasons, and an anonymized winner label. To reduce identity and position bias, model names were removed from the judge prompt, and the three responses for each question were randomly assigned to Answer A, Answer B, and Answer C. The private answer-label-to-model mapping was used only after scoring to aggregate model-level results. The raw prompt included six scored dimensions, including clarity/conciseness. For the manuscript-level quantitative comparison, the final reported weighted score was recalculated from the five domain-relevant dimensions only, after excluding clarity/conciseness as an auxiliary diagnostic indicator. The recalculated score followed the normalized formula reported in the main text: Overall score = 0.278D + 0.222A + 0.222S + 0.167T + 0.111L. This recalculation was performed using the stored dimension-level scores in the anonymized judge-score table. Therefore, the benchmark results should be interpreted as anonymized and shuffled automatic comparative evaluation results under the specified LLM-as-a-judge protocol, supported by a supplementary stratified 30-question blinded manual assessment rather than as absolute expert ratings.

Table S10d. Weighting sensitivity analysis record for GCMembraneBench.

Item	Setting
Input judge-score file	judge_scores_qwen_testset23_anonymized_shuffled.csv
Analysis output file	weight_sensitivity_analysis.csv

Item	Setting
Analysis purpose	To examine whether the model ranking was sensitive to the weighting scheme used for the five domain-relevant dimensions.
Tested weighting schemes	Reported domain-oriented weights, equal weights, technical-accuracy-emphasized weights, practical-usefulness-emphasized weights, and structure–performance-reasoning-emphasized weights.
Reported interpretation	The ranking of the three models remained unchanged under the tested weighting schemes.

The weighting sensitivity analysis was conducted using the anonymized and shuffled judge-score table. The detailed score values under each weighting scheme are provided in weight_sensitivity_analysis.csv.

Table S10e. Question-level textual isolation check between GCMembraneBench and the retained SFT QA dataset.

Item	Result
Retained SFT QA records checked	12,208
Benchmark questions checked	100
Exact textual duplicates	0
Very high textual overlap cases	0
Partial textual overlap cases	2
Questions with no direct textual duplication detected	98
Mean maximum character n-gram TF-IDF cosine similarity	0.426
Maximum character n-gram TF-IDF cosine similarity	0.6549
Mean maximum SequenceMatcher similarity	0.5113
Maximum SequenceMatcher similarity	0.7862

The check was designed to identify direct textual duplication and high wording overlap between the 100 benchmark questions and the complete set of 12,208 retained SFT QA records. Exact duplicates were defined after text normalization. Very high textual overlap was flagged when the character n-gram TF-IDF cosine similarity or SequenceMatcher similarity was at least 0.90, and partial textual overlap was flagged when either metric was at least 0.75. This analysis did not assess paper-level source isolation or semantic leakage because the benchmark records did not contain source-paper identifiers or DOI fields.

File S10b. Train–test textual isolation check between the 100-question GCMembraneBench and the complete 12,208-record retained SFT QA dataset.

train_test_isolation_check_100_questions_full_12208.xlsx

Table S10f. Category-level summary of the train–test textual isolation check.

Category	Number of benchmark questions	Exact duplicates	Very high overlap	Partial overlap	No direct duplication
CNT membrane	20	0	0	0	20
Application-condition and performance evaluation	15	0	0	1	14
GO/CNT hybrid membrane design	20	0	0	1	19
GO membrane applications and mechanisms	20	0	0	0	20
Application-oriented design and troubleshooting	10	0	0	0	10
Cross-material structure–performance evaluation	15	0	0	0	15

Table S10g. Supplementary stratified blinded manual assessment protocol.

Item	Setting
Source benchmark	GCMembraneBench
Number of sampled questions	30
Sampling strategy	Five questions sampled from each of the six benchmark categories
Compared models	GCMembrane-LLM, Llama-3.1-8B-Instruct, Doubao-1.5-lite
Blinding strategy	Model responses were presented using anonymized answer labels
Score scale	1 to 5
Reported metrics	Mean manual score and fractional win count
Interpretation	Supplementary human robustness check for the anonymized and shuffled automatic 100-question evaluation

Table S10h. Model-level results of the supplementary stratified blinded manual assessment.

Model	Mean manual score	Fractional wins
GCMembrane-LLM	4.060	14.33
Llama-3.1-8B-Instruct	4.013	11.83
Doubao-1.5-lite	3.840	3.83

Table S10i. Model-level results of the anonymized and shuffled 100-question automatic evaluation.

Model	Mean weighted score	95% confidence interval	Fractional wins

Model	Mean weighted score	95% confidence interval	Fractional wins
GCMembrane-LLM	4.237	4.087 to 4.378	62.5
Llama-3.1-8B-Instruct	3.896	3.778 to 4.012	33.5
Doubao-1.5-lite	2.845	2.723 to 2.968	4.0

Mean weighted scores were calculated from five domain-relevant dimensions using the normalized weighting formula reported in the main text. Confidence intervals were estimated by bootstrap resampling with 10,000 iterations. Fractional wins account for tied winners among model responses.

Table S10j. Paired score differences from the anonymized and shuffled 100-question automatic evaluation.

Paired comparison	Mean paired difference	95% confidence interval	Interpretation
GCMembrane-LLM minus Llama-3.1-8B-Instruct	0.341	0.143 to 0.533	Positive and stable
GCMembrane-LLM minus Doubao-1.5-lite	1.392	1.177 to 1.596	Positive and stable
Llama-3.1-8B-Instruct minus Doubao-1.5-lite	1.052	0.897 to 1.202	Positive and stable

Paired differences were calculated at the question level using the weighted overall score for the same benchmark question. A positive value indicates that the first model in the comparison achieved a higher mean score than the second model.

File S10c. Stratified 30-question blinded manual assessment workbook with sampled questions, anonymized model responses, and scoring rubric.

stratified_manual_blind_30_questions.xlsx

File S10d. Final score-only table for the stratified 30-question blinded manual assessment.

stratified_manual_blind_30_questions_final_scores_only.xlsx

File S5. Complete 100-question GCMembraneBench benchmark.

testset23_100_application.jsonl

File S5a. Category distribution of GCMembraneBench.

benchmark_category_distribution.csv

File S6. Responses generated by GCMembrane-LLM.

ours_testset23_100_results_long.csv

File S7. Responses generated by the original Llama-3.1-8B-Instruct model.

lama0_testset23_100_results.csv

File S8. Responses generated by Doubao-1.5-lite through the online inference endpoint.

doubao_testset23_100_results.csv

File S9. Anonymized and shuffled benchmark-answer table for the 100-question automatic evaluation.

anonymized_benchmark_answers_for_judge.csv

File S9a. Private answer-label mapping table used after scoring for model-level aggregation.

anonymized_answer_mapping_private.csv

File S10. Qwen-3.5-plus judge scores from the anonymized and shuffled 100-question automatic evaluation.

judge_scores_qwen_testset23_anonymized_shuffled.csv

File S10a. Model-level summary from the anonymized and shuffled 100-question automatic evaluation.

judge_scores_qwen_testset23_anonymized_shuffled_summary.csv

File S10b. Bootstrap confidence intervals for model-level scores and paired score differences.

confidence_interval_summary_anonymized_shuffled.csv

File S10c. Question-level paired score differences used for bootstrap confidence-interval analysis.

bootstrap_difference_results_anonymized_shuffled.csv

File S10d. Weighting sensitivity analysis results for GCMembraneBench.

weight_sensitivity_analysis.csv

File S10e. Train–test textual isolation check between the 100-question GCMembraneBench and the complete 12,208-record retained SFT QA dataset.

train_test_isolation_check_100_questions_full_12208.xlsx

File S10f. Stratified 30-question blinded manual assessment workbook with sampled questions, anonymized model responses, and scoring rubric.

stratified_manual_blind_30_questions.xlsx

File S10g. Final score-only table for the stratified 30-question blinded manual assessment.

stratified_manual_blind_30_questions_final_scores_only.xlsx

Code S11. GCMembrane-LLM benchmark response generation on the development instance.

Code_S10_GCMembrane_LLM_response_generation.py

Code S11a. Python script for calculating the GCMembraneBench category distribution.

Benchmark.py

Code S12. Original Llama-3.1-8B-Instruct benchmark response generation on the same development instance.

Code_S11_Llama_base_response_generation(1).py

Code S13. Doubao-1.5-lite benchmark response generation through the Volcengine Ark inference endpoint.

Doubao-1.5-lite.py

Code S14. Python script for anonymized and shuffled Qwen-3.5-plus automatic judging, answer-label randomization, JSON parsing, score aggregation, and bootstrap analysis.

run_anonymized_shuffled_100_judge.py

Code S15. Bootstrap confidence interval and paired-difference analysis for model comparison.

Bootstrap confidence interval and paired-difference analysis.py

Code S15a. Python script for GCMembraneBench weighting sensitivity analysis.

weighting sensitivity analysis.py

File S11. Full RAG records for Figure 5 and the three representative case studies.

all_rag_case_records.json

File S12. Figure 5: Source-grounding materials.

figure5_high_flux_interpretation_full_record.json
figure5_high_flux_interpretation_gcmembrane_rag_answer.txt
figure5_high_flux_interpretation_retrieval_records.csv

File S13. Retrieved evidence records and GCMembrane-LLM output for Case 1: GO/CNT composite membranes.

case1_gocnt_transport_selectivity_full_record.json
case1_gocnt_transport_selectivity_gcmembrane_rag_answer.txt
case1_gocnt_transport_selectivity_retrieval_records.csv

File S14. Retrieved evidence records and GCMembrane-LLM output for Case 2: GO membrane swelling and salt rejection.

case2_go_swelling_salt_rejection_full_record.json
case2_go_swelling_salt_rejection_gcmembrane_rag_answer.txt
case2_go_swelling_salt_rejection_retrieval_records.csv

File S15. Retrieved evidence records and GCMembrane-LLM output for Case 3: CNT membrane high flux and ion exclusion.

case3_cnt_flux_ion_exclusion_full_record.json
case3_cnt_flux_ion_exclusion_gcmembrane_rag_answer.txt
case3_cnt_flux_ion_exclusion_retrieval_records.csv

Code S16. Representative RAG case-study generation script.

run_all_rag_v4_final (1).py

Supplementary Abbreviations

BF16	Brain Floating Point 16-Bit Format
PEFT	Parameter-Efficient Fine-tuning
YAML	YAML Ain't Markup Language
